# Living donor liver transplantation for hepatocellular carcinoma beyond the Milan criteria: outcome of expanded criteria in tumor size

**DOI:** 10.1186/s12893-021-01403-z

**Published:** 2021-11-19

**Authors:** Hsin-Rou Liang, Chia-En Hsieh, Kuo-Hua Lin, Chih-Jan Ko, Yu-Ju Hung, Ya-Lan Hsu, Yao-Li Chen

**Affiliations:** 1 Department of General Surgery, Changhua Christian Hospital, No. 135 Nanxiao St., Changhua, 500 Taiwan (R.O.C.); 2 Department of Nursing, Changhua Christian Hospital, No. 135 Nanxiao St., Changhua, 500 Taiwan (R.O.C.)

**Keywords:** Living donor liver transplantation, Hepatocellular carcinoma, Beyond the Milan criteria

## Abstract

**Background:**

The Milan criteria are the universal standard of liver transplantation for hepatocellular carcinoma (HCC). Numerous expanded criteria have shown outcomes as good as the Milan criteria. In Taiwan, living donor liver transplant (LDLT) accounts for the majority of transplantations due to organ shortages.

**Methods:**

We retrospectively enrolled 155 patients who underwent LDLT for HCC from July 2005 to June 2017 and were followed up for at least 2 years. Patients beyond the Milan criteria (n = 78) were grouped as recurrent or nonrecurrent, and we established new expanded criteria based on these data.

**Results:**

Patients beyond the Milan criteria with recurrence (n = 31) had a significantly larger maximal tumor diameter (4.13 ± 1.96 cm versus 6.10 ± 3.41 cm, p = 0.006) and total tumor diameter (7.19 ± 4.13 cm versus 10.21 ± 5.01 cm, p = 0.005). Therefore, we established expanded criteria involving maximal tumor diameter ≤ 6 cm and total tumor diameter < 10 cm. The 5-year survival rate of patients who met these criteria (n = 134) was 77.3%, and the 5-year recurrence rate was 20.5%; both showed no significant differences from those of the Milan criteria. Under the expanded criteria, the pool of eligible recipients was 35% larger than that of the Milan criteria.

**Conclusion:**

Currently, patients with HCC who undergo LDLT can achieve good outcomes even when they are beyond the Milan criteria. Under the new expanded criteria, patients can achieve outcomes as good as those with the Milan criteria and more patients can benefit.

## Background

According to the World Health Organization (WHO), hepatocellular carcinoma (HCC) was the fourth most common cause of cancer death in the world in 2018. Hepatectomy is considered to be a curative treatment for HCC, but only 20–30% of HCCs are resectable at the time of diagnosis confirmation. In addition to anatomy-related unresectable HCC, some HCC patients suffer from inadequate remnant liver function due to chronic hepatitis virus infection or end-stage liver disease. In addition, many patients with HCC experience cancer recurrence after liver resection, which is the main cause of death in long-term follow-up. Liver transplantation (LT) can cure end-stage liver disease and remove any unresectable tumors and the part of the organ at high-risk of developing further malignancy. Accordingly, LT provides a significantly lower recurrence rate than hepatectomy.

The Milan criteria are the universally applicable standard of LT for HCC based on their significantly good outcome [1]. In recent years, there have been many published expanded criteria showing outcomes as good as that with the Milan criteria, such as UCSF criteria [2], up to seven [3] and others [4, 5]. The conventional criteria, especially the Milan criteria, have been criticized for being too strict and for depriving certain subgroups of the chance to benefit from transplant. The risk factors for posttransplantation HCC recurrence have also been discussed to create suitable expanded criteria for institutional facilities. There are some published risk factors, including time on the waiting list, model for end-stage liver disease (MELD) score, viral etiology, serum AFP level, maximal tumor diameter, vascular invasion, pretransplantation therapy and other factors [6–8], and some of these important factors are indeed included in the expanded criteria.

In Taiwan and other Asian countries, the shortage of organ resources has resulted in a prolonged waiting time, which can significantly affect the outcome of HCC. In addition, according to the Taiwan Organ Registry and Sharing Center, patients appearing on the waiting list for cadaveric liver grafts must meet the UCSF criteria. Therefore, living donor liver transplantation (LDLT) has become the major strategy for patients with HCC. We aim to present our experience with the outcomes of LDLT for HCC and to determine risk factors for posttransplantation recurrence and mortality in patients beyond the Milan criteria. We also established new and applicable expanded criteria based on the analysis of our patients, which may help physicians and institutions with patient selection and save more lives.

## Materials and methods

### Patients and study design

There were 346 adult LDLTs performed in Changhua Christian Hospital (a medical center in Taiwan) from July 2005 to June 2017. We included patients with (1) confirmed HCC before liver transplantation, (2) incidentally detected HCC in explant pathology, or (3) a previous history of HCC. Patients with HCC confirmed before LDLT were treated based on the Barcelona Clinic Liver Cancer (BCLC) guidelines first, such as radiofrequency ablation (RFA), surgical resection and transcatheter arterial chemoembolization (TACE). To ensure that the follow-up duration for HCC recurrence was adequate, we included patients who were followed up for at least 2 years after liver transplantation and excluded hospital mortalities. We retrospectively reviewed the electronic medical records of these HCC-associated recipients and recorded clinical factors, including age, MELD score, AFP, blood loss, HCC size, number, sex, imaging or pathology data, recurrence and mortality.

The patients were classified as either being within the Milan criteria or beyond the Milan criteria according to pretransplantation dynamic liver computed tomography (CT) imaging and explant pathology. We grouped the patients who were beyond the Milan criteria (based on explant pathology) into recurrence and recurrence-free groups and evaluated the important risk factors for HCC recurrence in these patients. After the new expanded criteria were derived, we regrouped all the patients as either meeting the new criteria or beyond the new criteria based on both imaging and pathology. The efficacy of the new criteria was evaluated by the survival rate and the HCC recurrence rate between patients within and beyond the new criteria. This study was approved by the Institutional Review Board (IRB) of Changhua Christian Hospital (IRB No. 191244).

### Pretransplantation evaluation

We routinely used dynamic liver CT, abdominal MRI with 3-dimensional reconstruction, chest CT and bone scans. If a suspicious lung nodule or bone hot spot was noted, the patient underwent lung or bone biopsy to rule out metastasis. Liver transplantation was not indicated in patients with confirmed extrahepatic metastasis. Other personal history, laboratory data, such as alpha-fetoprotein, and data for liver function evaluation were also evaluated.

### Posttransplantation follow-up

The regular follow-up for all recipients included alpha-fetoprotein level measurement and liver ultrasonography every 3 months and dynamic liver CT every 6 months. If the patient had no recurrence for 2 years after transplantation, the patient was then followed up with alpha-fetoprotein level measurement and liver ultrasonography every 6 months, and CT imaging of the liver or chest was performed once a year. None of the patients were lost to follow-up, except for mortality cases. HCC recurrence was diagnosed based on imaging evidence and biopsy proof. Once recurrent HCC was confirmed, the patient received various treatments, such as systemic chemotherapy, RFA, TACE, radiotherapy, and surgical resection, based on their performance status and tumor behavior.

### Liver CT image interpretation

Two physicians reviewed the pretransplantation liver CT images independently and recorded the maximal size of HCC, total tumor size of HCC, HCC number and vascular invasion. The image interpretation of those who had received local–regional therapy for HCC was based on the modified Response Evaluation Criteria in Solid Tumors (mRECIST) [9], which evaluates the viable (enhanced in the arterial phase) part of the lesions and excludes the necrotic or nonenhanced part.

### Explant liver pathologic interpretation

Final pathologic reports were based on the AJCC cancer staging manual, confirmed by one pathologic physician and peer reviewed by pathologic physicians and clinical doctors. The maximal size of HCC, total tumor size of HCC, HCC number and vascular invasion were recorded.

### Statistical analysis

Continuous variables are presented as the mean and standard deviation or the median with data range. The chi-square test or Fisher’s exact test (as appropriate) was conducted to compare categorical variables, and an independent *t* test was used to compare continuous variables between two groups. The cumulative recurrence rate and cumulative survival rate were compared by Kaplan–Meier survival curve analysis, and the difference between the two groups was assessed using the log-rank test. P values less than 0.05 were regarded as statistically significant. All statistical analyses were performed using SPSS software ver. 20.0 (IBM, Armonk, NY, USA).

## Results

There were 346 adult LDLTs performed in Changhua Christian Hospital (a medical center in Taiwan) from July 2005 to June 2017. During pretransplantation evaluation, 138 patients (39.9%) were diagnosed with HCC or suspected HCC, and 3 patients (0.8%) previously had HCC. HCC was incidentally detected via explant pathology in 14 patients (4%). These 155 patients were included in our analysis for posttransplantation HCC recurrence. The mean follow-up duration was 4.44 ± 2.35 years. The proportions of patients within the Milan criteria on the basis of pretransplant CT imaging and explant pathology were 63.9% and 49.7%, respectively. The compatibility rate between preoperative imaging and explant pathology was 83.2% (129 of 155 patients), and the overall demographic data and clinical features are listed in Table 1.

**Table 1:** Demographic data and clinical features of the patients enrolled in the analysis (n = 155)

	Overall (n = 155)	Range
	Mean ± SD (median)	
Male sex, n (%)	126 (81.3)	
Age (years)	56.21 ± 7.57 (56.00)	35.00–73.00
Diabetes mellitus, n (%)	31 (20)	
Etiology of cirrhosis, n (%)		
HBV	70 (45.2)	
HCV	53 (34.2)	
Alcoholic	13 (8.4)	
Alcoholic with HBV & HCV	6 (3.9)	
HBV and HCV	6 (3.9)	
Other	7 (4.5)	
MELD score	13.19 ± 7.30 (11.00)	5.00–40.00
Alpha-fetoprotein (ng/mL)	1524 ± 14,172.24 (16.66)	0.97–170,961.00
GRWR	1.07 ± 0.28 (1.02)	0.55–2.02
Operative time (min)	423.43 ± 87.44 (415.00)	265.00–690.00
Blood loss (ml)	2939.03 ± 3238.67 (1850.00)	200.00–23,000.00
Tumor characteristic		
Image tumor number	2.11 ± 2.18 (1.0)	0.00–10.00
Image max tumor size (cm)	2.83 ± 2.24 (2.3)	0.00–14.50
Image total tumor size (cm)	4.53 ± 4.21 (3.2)	0.00–25.40
Image vascular invasion, n (%)	13 (8.4)	
Pathology tumor number	2.54 ± 2.19 (2.0)	0.00–14.00
Pathology max tumor size (cm)	3.53 ± 2.50 (3.0)	0.00–16.10
Pathology total tumor size (cm)	5.62 ± 4.45 (4.0)	0.00–22.80
Pathology vascular invasion, n (%)	24 (15.5)	
Combined cholangiocarcinoma, n (%)	7 (4.5)	
Image and pathology compatible, n (%)	129 (83.2)	
Image—Met Milan criteria, n (%)	99 (63.9)	
Image—beyond Milan criteria, n (%)	56 (36.1)	
Pathology—Met Milan criteria, n (%)	77 (49.7)	
Pathology—beyond Milan criteria, n (%)	78 (50.3)	
Outcome		
HCC recurrent, n (%)	38 (24.5)	
Mortality, n (%)	44 (28.4)	
Follow up (year)	4.44 ± 2.35 (4.20)	0.27–12.09

*MELD* model for end-stage liver disease, *GRWR* graft-to-recipient weight ratio, *HBV* hepatitis B virus, *HCV* hepatitis C virus, *HCC* hepatocellular carcinoma

Thirty-eight of 155 patients (24.5%) had HCC recurrence events, and 29 of them had recurrence within 2 years after surgery. Thirty-one of them died from HCC recurrence. These patients with recurrence received different treatments for HCC recurrence according to their tumor behavior and performance status. The mean time between confirmed HCC recurrence and mortality was 1.10 ± 1.12 years (median 0.70, range 0.01–4.90 years).

### New expanded pathology-based criteria for LDLT in HCC

To evaluate the outcomes and risk factors of patients in the beyond the Milan criteria group (n = 78), we analyzed the clinical features of patients in the pathology-based beyond the Milan criteria group (Table 2). The patients beyond the Milan criteria with HCC recurrence events had a significantly larger maximal tumor size (mean ± SD: 6.10 ± 3.41 cm versus 4.13 ± 1.96 cm, p = 0.006), larger total tumor size (10.21 ± 5.01 cm versus 7.19 ± 4.13 cm, p = 0.005), and higher mortality rate (87.9% versus 12.1%, p < 0.001). There was no significant difference between recurrent and nonrecurrent tumors in other parameters, such as AFP level, tumor number and vascular invasion.

**Table 2:** Demographic data and clinical features of patients beyond the Milan criteria (n = 78) by explant pathology

	Recurrence-free (n = 47)		Recurrent (n = 31)		p value
	Mean ± SD	Range	Mean ± SD	Range	
Male sex, n (%)	41 (87.2)		30 (96.8)		0.233
Age (years)	56.21 ± 7.62	35–68	56.00 ± 7.37	43–72	0.903
MELD score	13.32 ± 7.20	5.0–35.0	11.58 ± 4.93	6.0–25	0.244
AFP (ng/mL)	4312.40 ± 25,171.44	2.49–170,961.00	687.37 ± 166.02	2.24–7889.00	0.434
Blood loss (ml)	2934.04 ± 3191.81	300.0–17,000.0	3870.97 ± 4941.79	200.0–23,000.0	0.312
Max HCC size (cm)	4.13 ± 1.96	1.10–10.00	6.10 ± 3.41	2.10–22.80	0.006
Total HCC size (cm)	7.19 ± 4.13	1.10–22.70	10.21 ± 5.01	2.10–22.80	0.005
HCC number	3.11 ± 1.88	0.0–9.0	4.29 ± 3.31	1.0–14.0	0.077
cHC-CC, n (%)	1 (2.1)		1 (2.1)		1.000
Vascular invasion, n (%)	11 (23.4)		13 (41.9)		0.083
AFP > 400 (ng/mL), n (%)	10 (21.3)		6 (19.4)		0.837
Mortality, n (%)	4 (8.5)		29 (93.5)		< 0.001

*MELD* model for end-stage liver disease, *AFP* alpha-fetoprotein, *HCC* hepatocellular carcinoma, *cHC-CC* combined hepatocellular-cholangiocarcinoma

According to these findings and outcomes, we set the eligibility criteria for HCC patients to receive LDLT as maximal tumor size ≤ 6 cm and total tumor size < 10 cm. A total of 134 of 155 patients met the new criteria by imaging, and 127 patients met the new criteria by pathology. The demographic data and clinical features of patients within and beyond the new criteria are listed in Table 3. Between patients within and beyond the new criteria, there were significant differences via explant pathology in maximal tumor size (p < 0.001), total tumor size (p < 0.001), tumor number (p = 0.001) and vascular invasion (p < 0.001). On the other hand, there were significant differences via pretransplantation imaging in maximal tumor size (p < 0.001), total tumor size (p < 0.001) and tumor number (p = 0.002). The impact of vascular invasion on HCC recurrence was unclear based on the discrepant results between imaging and pathology. In addition, we also confirmed the significant differences in mortality rate and recurrence rate between patients within and beyond the new criteria.

**Table 3:** Comparisons of the demographic data and clinical features of patients who met or were beyond the new criteria by imaging and pathology

Demographic and clinical features	Imaging determination			Pathology determination		
	Size ≤ 6 & < 10 cm (n = 134)	Size > 6 & ≥ 10 cm (n = 21)	p	Size ≤ 6 & < 10 cm (n = 127)	Size > 6 & ≥ 10 cm (n = 28)	p
	Mean ± SD (range)	Mean ± SD (range)		Mean ± SD (range)	Mean ± SD (range)	
Male sex, n (%)	107 (79.9)	19 (90.5)	0.369	100 (78.7)	26 (92.9)	0.109
Age (years)	56.18 ± 7.22 (35–73)	56.38 ± 9.71 (35–72)	0.928	56.43 ± 7.51 (35–73)	55.18 ± 7.92 (35–72)	0.429
MELD score	13.07 ± 7.40 (5.0–40.0)	13.95 ± 6.77 (6.0–35)	0.610	13.26 ± 7.45 (5.0–40.0)	12.89 ± 6.67 (6.0–35)	0.881
AFP (ng/mL)	258.80 ± 1008.63 (0.97–7224.41)	9501.08 ± 38,059.54 (4.09–170,961.00)	0.291	214.60 ± 905.04 (0.97–7224.41)	7046.68 ± 321.86.63 (4.09–170,961.00)	0.271
Blood loss (ml)	2779.48 ± 3008.75 (300.0–23,000.0)	3957.14 ± 4393.90 (200.0–16,000.0)	0.248	2874.02 ± 3153.83 (300.0–23,000.0)	3233.93 ± 3646.67 (200.0–16,000.0)	0.596
Max HCC size (cm)	2.25 ± 1.41 (0.0–5.8)	6.51 ± 3.01 (1.6–14.5)	< 0.001	2.76 ± 1.36 (0.0–6.0)	7.00 ± 3.43 (2.5–16.10)	< 0.001
Total HCC size (cm)	3.28 ± 2.36 (0.0–9.7)	12.50 ± 4.70 (6.6–25.4)	< 0.001	4.11 ± 2.55 (0.0–14.00)	12.45 ± 4.87 (6.20–22.8)	< 0.001
HCC number	1.74 ± 1.65 (0.0–10.0)	4.60 ± 3.44 (1.0–10.0)	0.002	2.16 ± 1.80 (0.0–14.0)	4.29 ± 2.88 (1.0–10.0)	0.001
cHC-CC, n (%)	6 (4.5)	1 (4.8)	1.000	7 (5.5)	0 (0)	0.352
Vascular invasion, n (%)	10 (7.5)	3 (14.3)	0.387	18 (14.2)	6 (21.4)	< 0.001
AFP > 400 (ng/mL), n (%)	11 (8.2)	7 (33.3)	< 0.001	9 (7.1)	9 (32.1)	< 0.001
Recurrent, n (%)	27 (20.1)	11 (52.4)	0.001	22 (17.3)	16 (57.1)	< 0.001
Mortality, n (%)	32 (23.9)	12 (57.1)	0.002	28 (22.0)	16 (57.1)	< 0.001

*MELD* model for end-stage liver disease, *AFP* alpha-fetoprotein, *HCC* hepatocellular carcinoma, *cHC-CC* combined hepatocellular-cholangiocarcinoma

### Comparison of survival outcome and HCC recurrence rate based on the new criteria

The cumulative recurrence rates and survival rates were not significantly different between image-based and pathology-based determinations (Table 4). This result indicates that the differences between imaging and pathology under the new criteria have no significant influence on predicting outcome.

**Table 4:** Comparisons of HCC recurrence rate and patient survival rate by imaging and pathology

	Imaging Size ≤ 6 & < 10 cm (n = 134)	Pathology Size ≤ 6 & < 10 cm (n = 127)	p	Imaging Size > 6 & ≥ 10 cm (n = 21)	Pathology Size > 6 & ≥ 10 cm (n = 28)	p
HCC recurrence rate			0.518			0.580
1 Year	9.8	5.5		23.8	39.3	
2 Years	15.8	11.9		38.1	50.0	
3 Years	18.4	15.5		43.3	54.2	
5 Years	20.5	17.8		56.2	59.3	
10 Years	25.2	22.7		56.2	59.3	
Patient survival rate			0.693			0.834
1 Years	95.5	97.6		100.0	89.3	
2 Years	91.0	93.7		76.2	67.9	
3 Years	83.1	86.1		65.6	37.9	
5 Years	77.3	79.3		36.1	37.9	
10 Years	64.3	65.6		36.1	37.9	

In addition, based on the cumulative recurrence rates and survival rates listed in Tables 3 and 4, significantly better outcomes were noted in patients within the new criteria than in those beyond the new criteria. The mean recurrence times were 4.42 ± 2.44 years and 2.94 ± 2.82 years (p = 0.006) for patients within the new criteria and for patients beyond the new criteria, respectively.

**Fig. 1:**
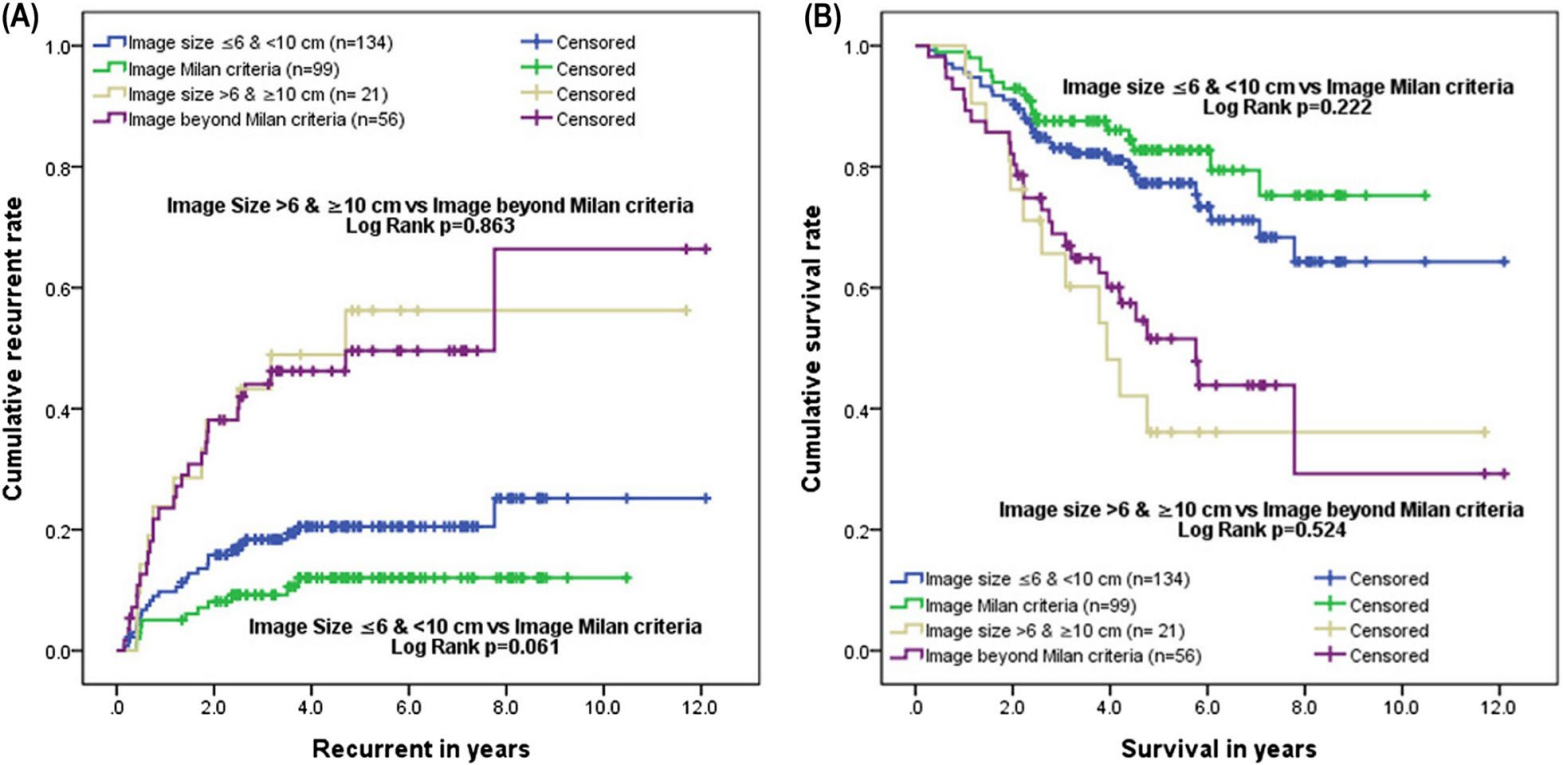
Comparison of cumulative survival rate and recurrence rate in patients within the Milan criteria and within the new expanded criteria based on the pretransplantation image. The cumulative recurrence rate (**A**) and cumulative survival rate (**B**) showed no significant difference between the Milan criteria and the new expanded criteria by pretransplantation imaging

Based on pretransplantation imaging, the 5-year survival rates were 82.4% and 77.3% for those who met the Milan criteria and those who met the new criteria, respectively; there was no significant difference between the two criteria (p = 0.222). Based on pretransplantation imaging, the 5-year recurrence rates were 12.0% and 20.5% for those who met the Milan criteria and those who met the new criteria, respectively; there was no significant difference between the two criteria (p = 0.061) (Fig. 1). For the survival rate and recurrence rate, the new criteria performed as well as the Milan criteria in terms of providing the outcome and predictive power.

## Discussion

The outcomes of patients beyond the Milan criteria showed various results in previous studies. Some studies showed that similar survival outcomes could occur between patients who met the Milan criteria and patients beyond the Milan criteria [6, 10]. There were many reports indicating that patients beyond the Milan criteria could have significantly good outcomes if they met certain conditions, and many expanded criteria have been presented, such as the UCSF criteria [2], Asan criteria [11], up-to-seven criteria [3], Kyushu criteria [12], and NCCK criteria [13]. This study included 155 patients with HCC who underwent LDLT from July 2005 to June 2017. Based on the outcome analysis of patients beyond the Milan criteria, we established new expanded criteria, which are maximal tumor size ≤ 6 cm and total tumor size < 10 cm. The 5-year survival rates were similar without significant differences among pretransplantation imaging in patients who met the Milan criteria, and pretransplantation imaging or pathology in patients who met the new criteria (82.4% versus 77.3% versus 79.3%, p = 0.471).

Sung-Gyu Lee et al. [11] suggested expanded criteria named the “Asan criteria” in 2008, which were largest tumor diameter ≤ 5 cm, HCC number ≤ 6, and no gross vascular invasion. Based on their study, the 5-year survival rate and 3-year recurrence rate in patients within the Asan criteria were 81.6% and 13%, respectively, which were not significantly different from those of patients who met the Milan criteria (76% and 13.6%) in their study. The Asan criteria (174 patients) expanded the pool of LDLT-eligible patients by 6%, and most of the patients within the Asan criteria also met the Milan criteria (164 patients). Our new criteria showed similar outcomes and included more patients than that used to establish the Asan criteria.

A multicenter retrospective study in Japan [14] proposed new criteria in 2019 called the “5-5-500 rule”, which were nodule size ≤ 5 cm in diameter, nodule number ≤ 5, and alpha-fetoprotein value ≤ 500 ng/ml. There were 664 patients within the Milan criteria and 735 patients within the 5-5-500 rule, which represented an 11% increase in the number of eligible patients. Among the patients who met the 5-5-500 rule, the 5-year overall survival rate was 75.8%, and the 5-year recurrence rate was 7.3% in their study.

Based on pretransplantation imaging, 99 patients met the Milan criteria, and 134 met the new criteria in our study. Under these new selection criteria, the candidate pool was expanded by 35%, and similar outcomes compared to the Milan criteria were reached (Fig. 1). Therefore, we believe this set of new criteria is as applicable as the Milan criteria. On the other hand, we also noted the very poor outcomes of patients beyond the new criteria; thus, LDLT is not recommended in patients beyond the new criteria (Table 4). The best treatment strategy for posttransplantation recurrence is still controversial, and the topic needs more research. Systemic adjuvant therapy might be available in the future, but more clinical trials are needed.

In Table 3, we compared the demographic data and clinical features of patients meeting and beyond the new criteria; there were significant differences in maximal tumor size, total tumor size, tumor number, AFP > 400 ng/ml, recurrence rate and mortality rate. The common risk factors that were discussed in previous studies [6] included AFP level, maximal tumor diameter, tumor number, macrovascular invasion, histological grade [15], distribution, and PIVKA-II [16]. We noted that the main difference and incompatibility between imaging and pathology was vascular invasion. Macrovascular invasion is currently not an absolute contraindication in LDLT [17], and microvascular invasion cannot be confirmed until explant pathology [18, 19]. In pretransplant images, there were 13 patients (8.4%) who had macrovascular invasion, and 6 of them had recurrent episodes, which were all extrahepatic metastases. However, in explant pathology, there were almost twice as many patients (24 patients, 15.5%) who had vascular invasion.

We aimed to set widely available and noninvasive criteria to maintain good outcomes and expand the number of candidates. Our new criteria are maximal tumor diameter ≤ 6 cm, total tumor diameter < 10 cm and no extrahepatic metastasis. The new criteria abandoned tumor number and vascular invasion because in our study, tumor number and vascular invasion were not significantly different between the recurrence and nonrecurrence groups of patients beyond the Milan criteria via explant pathology (Table 2). In recently published expanded criteria, tumor number [6] and vascular invasion were ignored, and more focus was given to maximal tumor size and other parameters, such as histological differentiation [20] and PET scan [13, 21].

This study has some limitations. First, this was a retrospective study; thus, it inevitably contains inaccuracies and selection bias. Therefore, further prospective studies to evaluate the new criteria are needed. Second, patients who underwent LDLT for HCC in our hospital had relatively lower AFP levels than the general HCC patients. In our study, 77.4% of patients had AFP levels below 400 ng/ml. Because high AFP levels and macrovascular invasion have been suggested to be risk factors for posttransplant HCC recurrence in previous studies [15, 16], we tended to turn down these high-risk patients for LDLT. This selection bias may explain why the AFP level and vascular invasion were not significant risk factors in our study. In addition, vascular invasion was not a significant risk factor for recurrence in patients beyond the Milan criteria (Table 2), but the vascular invasion rate in explant pathology was almost double in the recurrent group, and the p value was 0.083. This may result from a relatively small sample and the difficulty in diagnosing microvascular invasion by imaging.

## Conclusion

Currently, select patients with HCC undergoing LDLT can have good outcomes even if they are beyond the Milan criteria. We presented new expanded criteria: maximal tumor diameter ≤ 6 cm, total tumor diameter < 10 cm and no extrahepatic metastasis. Under the expanded criteria, patients can achieve good outcomes and more patients can benefit.

## Data Availability

All data generated or analysed during this study are included in this published article.

## Abbreviations

HCC: Hepatocellular carcinoma
MELD score: Model for end-stage liver disease score
cHC-CC: Combined hepatocellular-cholangiocarcinoma
LDLT: Living donor liver transplant
LT: Liver transplantation
RFA: Radiofrequency ablation
TACE: Transcatheter arterial chemoembolization
CT: Computed tomography
